# Intranasal acellular pertussis vaccine provides mucosal immunity and protects mice from *Bordetella pertussis*

**DOI:** 10.1038/s41541-019-0136-2

**Published:** 2019-10-03

**Authors:** Dylan T. Boehm, M. Allison Wolf, Jesse M. Hall, Ting Y. Wong, Emel Sen-Kilic, Hayden D. Basinger, Sebastian A. Dziadowicz, Maria de la Paz Gutierrez, Catherine B. Blackwood, Shelby D. Bradford, Katherine A. Begley, William T. Witt, Melinda E. Varney, Mariette Barbier, F. Heath Damron

**Affiliations:** 1 Department of Microbiology, Immunology, and Cell Biology, West Virginia University, Morgantown, WV USA; 2 Vaccine Development center at West Virginia University Health Sciences Center West Virginia University, Morgantown, WV USA; 3 West Virginia University School of Medicine, Morgantown, WV USA; 4 Instituto de Biotecnología y Biología Molecular (IBBM)-CCT-CONICET-La Plata, Departamento de Ciencias Biológicas, Facultad de Ciencias Exactas, Universidad Nacional de La Plata, La Plata, Argentina

**Keywords:** Vaccines, Protein vaccines, Bacterial infection, Adjuvants, Immunology

## Abstract

Current acellular pertussis vaccines fall short of optimal protection against the human respiratory pathogen *Bordetella pertussis* resulting in increased incidence of a previously controlled vaccine- preventable disease. Natural infection is known to induce a protective mucosal immunity. Therefore, in this study, we aimed to use acellular pertussis vaccines to recapitulate these mucosal immune responses. We utilized a murine immunization and challenge model to characterize the efficacy of intranasal immunization (IN) with DTaP vaccine or DTaP vaccine supplemented with curdlan, a known Th1/Th17 promoting adjuvant. Protection from IN delivered DTaP was compared to protection mediated by intraperitoneal injection of DTaP and whole-cell pertussis vaccines. We tracked fluorescently labeled DTaP after immunization and detected that DTaP localized preferentially in the lungs while DTaP with curdlan was predominantly in the nasal turbinates. IN immunization with DTaP, with or without curdlan adjuvant, resulted in anti-*B. pertussis* and anti-pertussis toxin IgG titers at the same level as intraperitoneally administered DTaP. IN immunization was able to protect against *B. pertussis* challenge and we observed decreased pulmonary pro-inflammatory cytokines, neutrophil infiltrates in the lung, and bacterial burden in the upper and lower respiratory tract at day 3 post challenge. Furthermore, IN immunization with DTaP triggered mucosal immune responses such as production of *B. pertussis*-specific IgA, and increased IL-17A. Together, the induction of a mucosal immune response and humoral antibody-mediated protection associated with an IN administered DTaP and curdlan adjuvant warrant further exploration as a pertussis vaccine candidate formulation.

## Bordetella pertussis: mimicking the mucosal response for protection

An acellular pertussis vaccine is used to protect against whooping cough, caused by the bacterial pathogen *Bordetella pertussis*; however, there is a growing number of pertussis cases despite vaccination. Here Dylan Boehm and colleagues combine the acellular vaccine with a pro-inflammatory adjuvant to maximise the mucosal immune response when administered intra-nasally in a murine model. They use curdlan, a 1,3 β-glucan, which is known for its Th1/Th17 promoting abilities. Intra-nasal vaccination with curdlan resulted in protection that was comparable to typical intra-peritoneal administration or whole-cell pertussis vaccines, with similar IgG levels against the bacterium and its toxin. However, intra-nasal administration with curdlan resulted in the vaccine localizing in the nasal turbinates rather that the lungs. When used in a challenge model, intra-nasal immunization reduced bacterial burden in the upper and lower respiratory tract, decreased pro-inflammatory cytokines and immune cell infiltrates, and triggered *B. pertussis*-specific IgA and IL-17A production, suggesting that this vaccination route and adjuvant may warrant further studies.

## Introduction

Pertussis is a human upper respiratory disease, primarily caused by the Gram-negative pathogen *Bordetella pertussis*. The disease is most severe in unvaccinated infants, where it manifests as fits of paroxysmal coughs followed by the classical whoop as air re-enters the respiratory system, leading to the more commonly known name: whooping cough.^1^ Additional complications include vomiting, increased mucus production, and apneic episodes. In severe cases, these symptoms can lead to elevated leukocytosis, pulmonary hypertension, hypoxia, and in some cases death.^1–3^ Furthermore there is speculation that pertussis may enhance development of neurodegenerative diseases later in life.^4^

In the pre-vaccine era, pertussis was responsible for 200,000 deaths annually in the United States.^5^ Widespread use of diphtheria tetanus whole-cell pertussis (DTP; wP) vaccines controlled the incidence of pertussis.^6^ However, adverse side effects associated with wP immunization^1^ led to the development of an acellular pertussis vaccine (DTaP; aP) that was introduced into the schedule in 1996. In the United States the number of pertussis cases has been increasing since the 1990s, despite high vaccine coverage.^7^ Additionally, increasing numbers of cases have been documented in older children, adolescents, and adults.^8^ The increase in incidence of pertussis cases suggests that current aP vaccines do not offer complete protection from *B. pertussis* infection. However, they do prevent fatal cases of pertussis as aP immunized individuals have low death rates.^8^ Multiple hypotheses have been proposed explaining the resurgence of pertussis cases including: (1) waning of protective immunity from DTaP/Tdap,^9–11^ (2) vaccine driven evolution of *B. pertussis* strains,^12^ (3) the possibility of increased transmission through asymptomatic carriers,^13^ and (4) increased surveillance and more accurate diagnoses technology.

Vaccine-induced protection in aP immunized individuals has been associated with a robust antigen-specific IgG response to the components of the aP vaccines.^14–17^ Likewise, wP immunization also resulted in antigen-specific IgG responses; with the addition of a shift to a more diverse T cell response, inducing cell-mediated immunity.^18^ In the murine model, immunization through intramuscular (IM) and intraperitoneal (IP) administration has been well characterized demonstrating a Th1/Th17 response from wP immunized mice, and a Th2 with weak Th17 mediated response in aP immunized mice following *B. pertussis* challenge.^19^ However, these immunizations fail to induce the mucosal immune responses elicited from natural infection. In murine challenge models recent studies have revealed that protection correlates with tissue resident memory T (T_RM_) cells in the lung and nasal cavity of convalescent mice, that produce interleukin-17 (IL-17) and interferon-gamma (IFN-γ), although T_RM_ activity in pertussis is yet to be studied in humans.^20^ T_RM_ cells have been shown to persist in the respiratory tissue and expand upon re-challenge of a convalescent mouse with *B. pertussis*, as well as decrease bacterial burden upon adoptive transfer to naive mice.^21^ More recently the expansion of this population has been observed following immunization by wP, a live-attenuated wP vaccine, an outer membrane vesicle vaccine, and intranasal administration of an aP vaccine with TLR9 and stimulator of interferon genes (STING) agonists.^22–25^ The study of this population has renewed interest in the induction of a mucosal immune response capable of decreasing bacterial burden at the site of infection.

The induction of a mucosal immune response to *B. pertussis* is associated with the production of secretory IgA antibodies (sIgA) in the nasal cavity. In humans previously infected with *B. pertussis*, IgA antibodies have been isolated from nasal secretions.^26^ *B. pertussis*-specific IgA antibodies isolated from convalescent patients have been shown to inhibit adherence of *B. pertussis* to respiratory epithelial cells in vitro,^27^ suggesting a protective role of IgA antibodies in mucosal immunity. Initial studies using IgA-deficient mice did not show strong support for a critical role in bacterial clearance of the respiratory tract.^28^ However, work with a live-attenuated IN pertussis vaccine (BPZE1) recently demonstrated protective role of sIgA antibodies in the respiratory tract.^24^

DTaP vaccine does not contain a strong pro-inflammatory adjuvant such as endotoxin of wP vaccine or BPZE1. We aimed to investigate IN DTaP immunization alone or with an additional pro-inflammatory adjuvant. We formulated vaccines containing the adjuvant curdlan, a 1,3 β-glucan, derived from *Alcaligenes faecalis*. This polysaccharide has immunostimulatory properties and forms a “sticky” gel at a neutral pH.^29^ Importantly, curdlan has been shown to bind to dendritic cells through the ligand Dectin-1, thereby inducing expression of NF-κB leading to a Th1/Th17 mediated immune response as well as production of antigen-specific respiratory IgA antibodies and serum IgG antibodies.^30–33^ Our objective was to determine whether IN DTaP immunization would induce a protective immune response in a murine model. We observed that the gel properties of curdlan facilitated DTaP localization in the upper respiratory tract. We determined a significant reduction in bacterial burden following administration of intranasally administered DTaP vaccines. We measured high serum and respiratory antibody responses produced, following intranasal administration of DTaP with and without curdlan. This study suggests mucosal vaccination with acellular vaccine may be a strategy for decreasing incidence of pertussis.

## Results

### Acellular pertussis vaccine was retained in the upper and lower respiratory tract when administered by intranasal administration

**Fig. 1:**
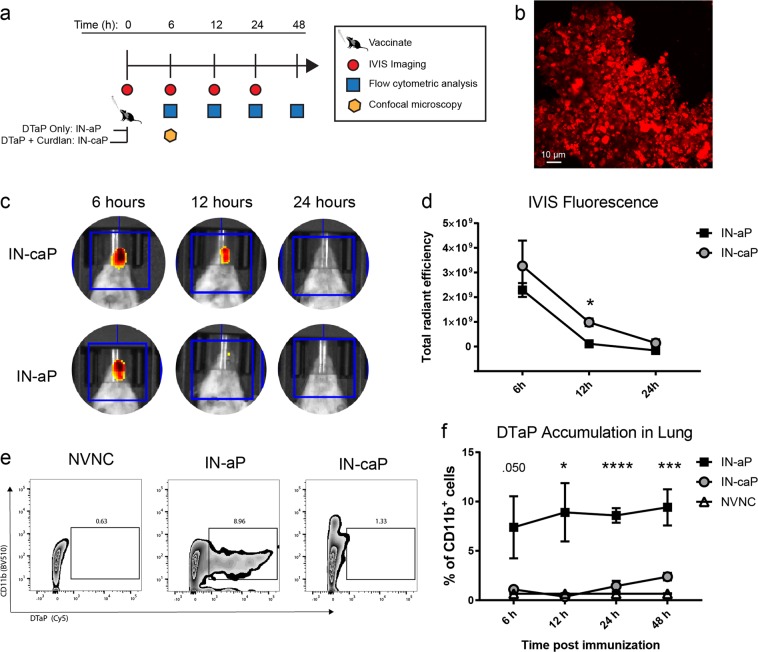
Localization of acellular pertussis vaccine in the upper and lower respiratory system after IN vaccination. **a** Schematic of vaccine tracking protocol. CD-1 mice were intranasally vaccinated with either fluorescent DTaP alone (IN-aP) or fluorescent DTaP with curdlan (IN-caP). Vaccine particle deposition in the lungs and nasal cavity was measured at 0, 6, 12, 24, and 48 h after immunization. **b** Representative image of Alexa Fluor labeled DTaP vaccine particles. **c** Representative images of nasal cavity fluorescence at 6, 12, and 24 h. The region of interest used for fluorescence quantification is shown in blue. (**d**) Fluorescence measurements normalized to PBS control at 6, 12, and 24 h (*n* = 4). Results shown as mean ± SEM of total radiant efficiency, *P *<* 0.05. *P* values were determined by multiple *T*-tests with Holm-Sidak post hoc test between IN-aP and IN-caP vaccinated mice. **e** Representative plots at 12 h showing live, single cells that are CD11b^+^DTaP^+^. **f** Flow cytometric analysis of CD11b^+^ cells from the lung that contain or are bound to DTaP particles at 6, 12, 24, and 48 h post immunization. Results shown as mean ± SEM, *P *<* 0.05, ***P *<* 0.001, ****P *<* 0.0001 (*n* = 4). *P* values were determined by one-way ANOVA with Dunnett’s post hoc test comparing IN-aP immunized mice to control mock vaccinated mice

Pertussis toxin (PT) is an essential virulence factor, responsible for multiple factors in the pathogenesis of *B. pertussis*. PT facilitates infection by aiding in adherence to ciliated airway epithelial cells and through disruption of host innate immune cell recruitment to the site of infection.^34–36^ In numerous studies it has been demonstrated that neutralization of PT alone ablates symptoms of the disease.^37–40^ Therefore, we proposed that neutralization of PT at the site of infection could inhibit the systemic long-range activity of PT before colonization of the respiratory tract. We hypothesized that intranasal immunization would prime a protective systemic and mucosal immune response. Furthermore, the gel-like properties of curdlan may have a beneficial role in increasing antigen uptake. To determine if use of curdlan would increase vaccine retention in the respiratory system, we IN vaccinated CD-1 mice with commercially available DTaP (IN-aP), DTaP with curdlan (IN-caP), or PBS (mock vaccinated) and tracked the vaccine up to 48 h after vaccination (Fig. 1a). To visualize vaccine presence in the respiratory system we labeled DTaP vaccine particles with a fluorophore (Fig. 1b). We measured the size of the labeled particles and determined that size was similar to what has been previously reported, and labeled particles were on average were 1.52 ± 0.76 µm. These findings are comparable to previous work which found that 90% of DTaP particles were between 1–3 µm.^41^ Using in vivo animal imaging, we observed fluorescently labeled DTaP particles in the nasal cavity at 6, 12, and 24 h post-vaccination (Fig. 1c). At 12 h post-vaccination, we detected significantly higher levels of fluorescence in IN-caP vaccinated mice compared to IN-aP mice (Fig. 1d), suggesting more DTaP particles were retained in the nasal cavity. This method resulted in the quantification of total particles in nasal cavity. To quantify DTaP particles that were bound to innate immune cells we utilized flow cytometry. Single-cell suspensions were prepared from homogenized lung tissue and antigen presenting cells (APCs) bound to DTaP were quantified as live, single cells positive for CD11b^+^DTaP^+^ (Fig. 1e). We observed a significant increase in CD11b^+^ cells that were bound to or contained DTaP particles in IN-aP mice compared to IN-caP (Fig. 1f). Together, these data suggest a higher deposition of DTaP in the lung with IN-aP when compared to IN-caP. Conversely, in the nasal cavity we measured higher levels of DTaP when mice were vaccinated with IN-caP, compared to IN-aP. These findings suggest that addition of curdlan to the DTaP vaccine causes retention in the nasal cavity, but without it, the vaccine components are more readily detected in the lung.

**Fig. 2:**
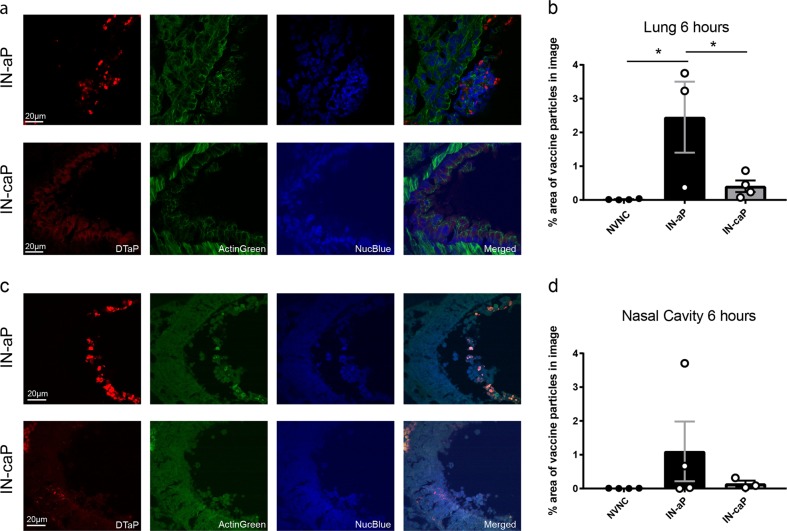
Acellular pertussis vaccine particle localization altered by curdlan adjuvant. **a** Representative images of flash frozen lung sections 6 h after immunization with IN-aP or IN-caP. Fluorescent particles were detected using a 660 laser, samples were counter-stained with NucBlue (blue) and ActinGreen (green). **b** Fluorescent DTaP particles were quantified by determining the percentage area of particles per field of view. (*n* = 3–4, with averages of three images per lung). Results are shown as mean ± SEM, *P *<* 0.05. **c** Representative images of paraffin embedded nasal cavity sections 6 h after immunization with IN-aP or IN-caP. **d** Fluorescent DTaP particles quantified by determining the percentage area of particles per field of view. (*n* = 3–4, with averages of three images per lung). *P* values were determined by one-way ANOVA with Tukey’s post hoc test

To visualize the deposition of DTaP particles, sections from the lung and nasal cavity were imaged using confocal microscopy. Vaccinated mice were euthanized after 6 h, lung tissue was flash frozen and skulls were embedded in paraffin for sectioning. Sections from the lung and nasal cavity were counterstained with NucBlue and ActinGreen to visualize epithelial tissue and fluorescent DTaP particles (Fig. 2a, c). Vaccine particles were quantified by measuring the percentage of total image field emitting DTaP fluorescence. We detected a significant increase of fluorescent particles in the lungs of mice that were vaccinated with IN-aP compared to IN-caP (Fig. 2b). Using microscopy, there was no significant difference in the number of particles detected in the nares comparing IN-aP to IN-caP (Fig. 2d). Interestingly, we observed that DTaP particles from the IN-aP vaccinated mice were localized in the lumen of the nasal passages, while particles from IN-caP vaccinated mice were deposited into the epithelial cells (Fig. 2c). Overall these data suggest curdlan impacts localization of DTaP in the airway.

### Intranasal immunization induces production of anti-PT and anti-FHA IgG in serum

To determine vaccine-induced protection, we utilized an established vaccine and challenge protocol.^42^ CD-1 mice were immunized IN with either IN-aP or IN-caP. These groups were compared to pertussis immunizations that are known to be protective: DTaP (IP-aP) and whole-cell pertussis (IP-wP) administered intraperitoneally (IP). Additionally, we aimed to determine if the effects of curdlan were dependent by route, thus we included mice immunized with IP delivered DTaP and curdlan (IP-caP). Lastly, we compared the findings to mock vaccinated mice, with IN delivered PBS, or mice IN immunized with curdlan only (IN-curdlan). All mice were immunized with 1/12th of the antigen load contained in the human vaccine dose. Mice were vaccinated at day 0, then received a booster vaccine at day 21. Mice were challenged on day 35 with *B. pertussis*.

**Fig. 3:**
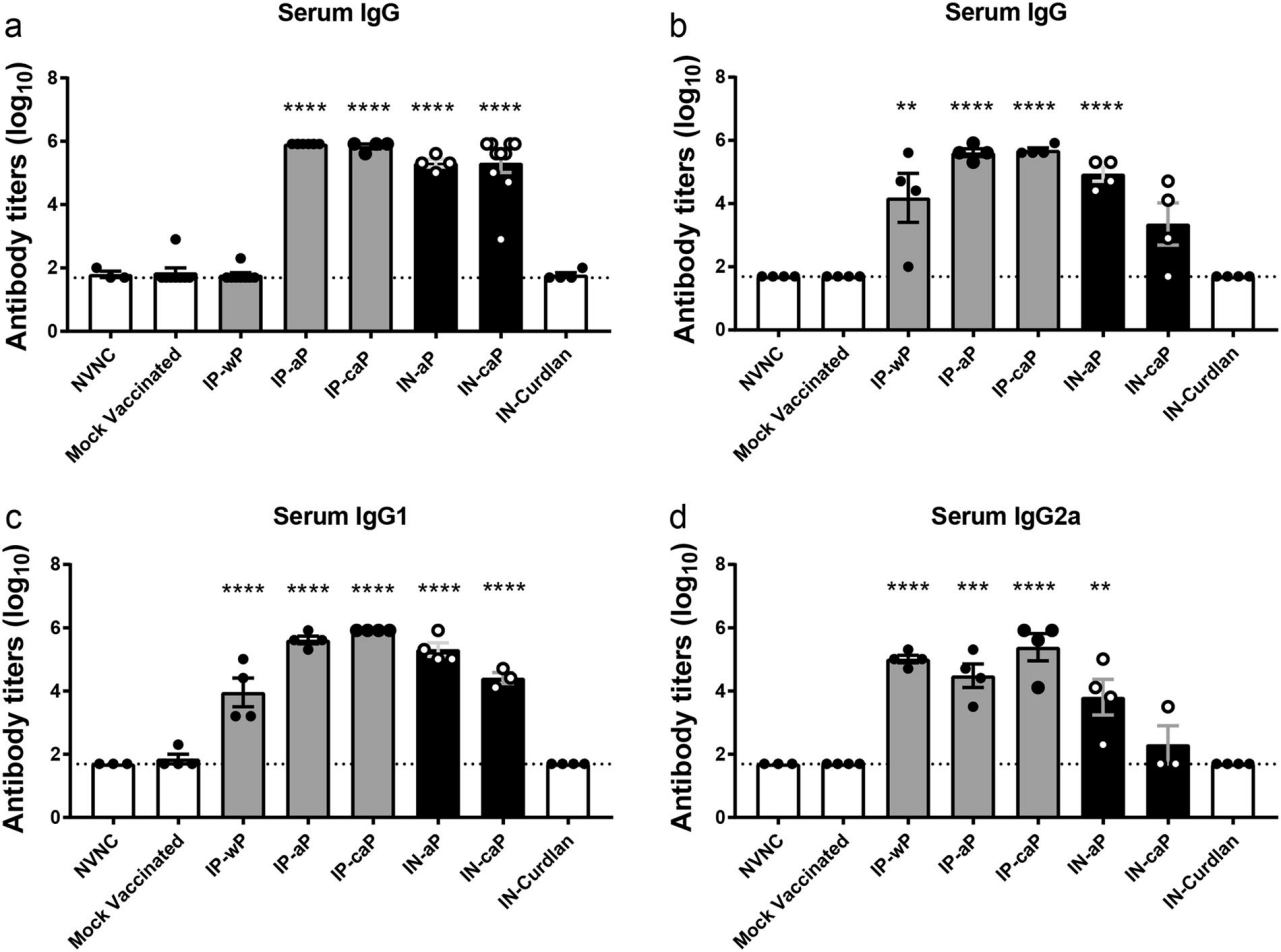
Intranasal immunization induces production of anti-PT and anti-FHA IgG in serum. ELISAs were used to compare serological responses from mice immunized through IN or IP routes to mock vaccinated mice. Total IgG serum antibody titers from immunized and challenged mice were quantified against **a** PT and **b** FHA at day 3 pc. Serum **c** IgG1 and **d** IgG2 antibody titers against *B. pertussis* were compared to mock vaccinated mice at day 3. Results shown as mean ± SEM, **P *<* 0.01, ***P *<* 0.001, ****P *<* 0.0001 (*n* = 3–8). *P* values were determined by one-way ANOVA with Dunnett’s post hoc test compared to mock vaccinated mice

We first sought to determine if an IN delivered DTaP vaccine would induce a systemic immune response. To answer this, we performed enzyme-linked immunosorbent assays (ELISAs) with serum from vaccinated and challenged mice against *B. pertussis* antigens found in the vaccine: PT and filamentous hemagglutinin (FHA). ELISAs were not performed against pertactin antigen, as 85% of current clinical isolates in the US do not express the protein.^43^ Serum anti-PT IgG titers were similar between mice immunized with IP-aP and those immunized through IN administration, as no significant differences were determined between IP-aP, IN-aP or IN-caP (Fig. 3a). Similarly, we observed a robust titer response to the bacterial adhesin FHA in IN vaccinated mice; however, IN-aP serum anti-FHA titer was sevenfold higher than IN-caP (Fig. 3b). We next determined if IN administration of DTaP (with or without curdlan) would lead to an increased Th1 immune response resulting in a higher ratio of IgG2a compared to IgG1 antibodies. Neither route nor adjuvant impacted the ratio of IgG2a to IgG1.

### Intranasal immunization induces production of anti-*B. pertussis* IgA in lung

**Fig. 4:**
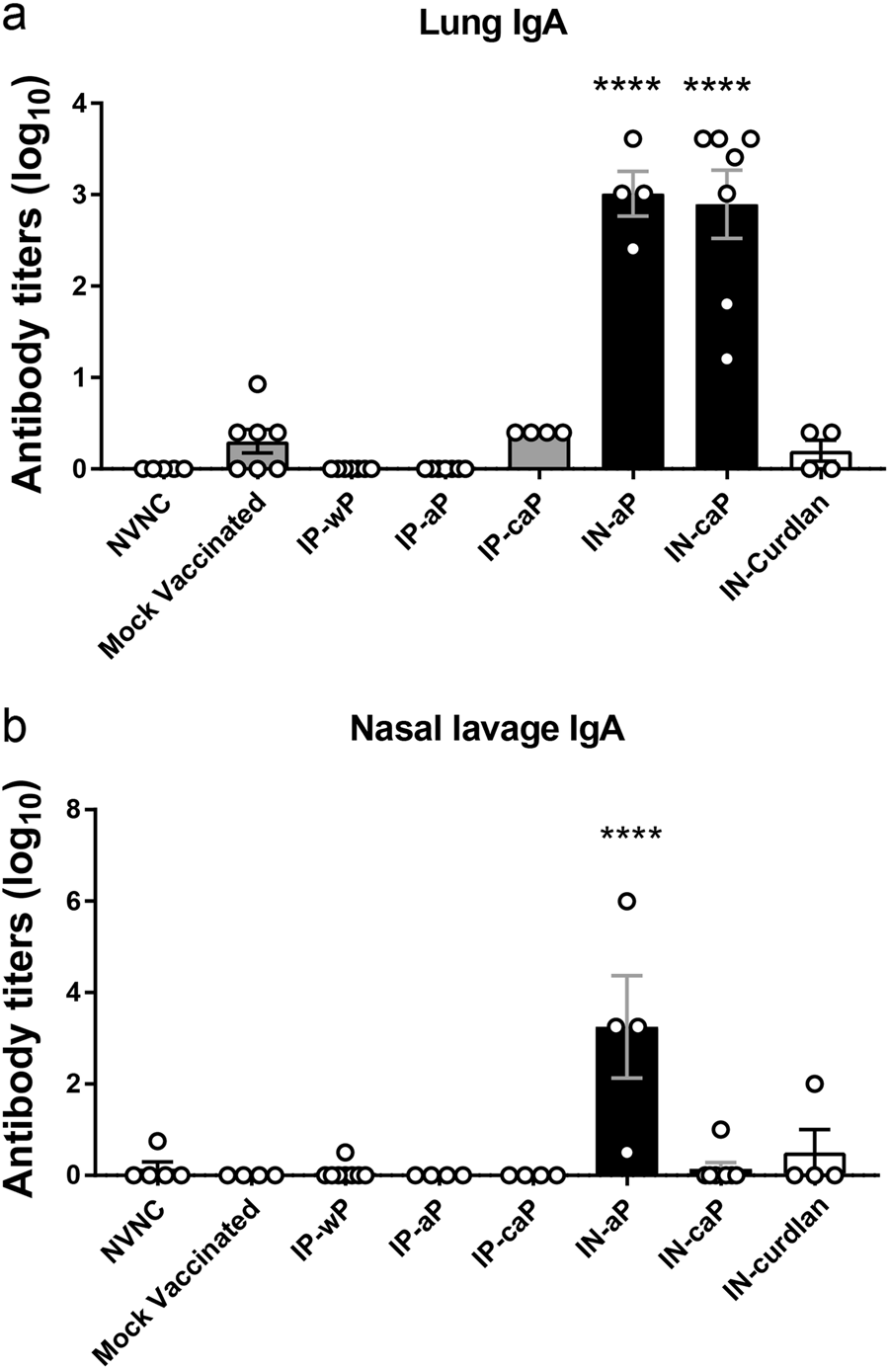
Intranasal immunization induces production of anti-*B. pertussis* IgA in respiratory system. ELISAs were performed using **a** lung homogenate supernatant and **b** nasal lavage fluid from vaccinated and challenged mice at day 3 pc. IgA titers were determined against whole-cell *B. pertussis*. Results are shown as averages of two independent experiments, represented on a log10 scale for lung and linear scale of nasal lavage with mean ± SEM (*n* = 4-8). ****P *<* 0.0001. *P* values were determined by one-way ANOVA with Dunnett’s post hoc test compared to mock vaccinated mice

In the murine model, recent data has pointed to the importance of IgA and a local mucosal immune response in the lung and nasal cavity.^21,22,24,25^ We aimed to measure the presence of IgA antibodies in the murine respiratory tissue due to IN immunization. Using ELISAs we measured *B. pertussis* specific IgA titers in homogenized lung tissue supernatant and nasal lavage fluid. Interestingly, we detected a robust IgA response in the lung only when mice were immunized through the IN route (Fig. 4a). Antigen-specific IgA response was not observed in mice immunized with curdlan alone. We observed detectable IgA *B. pertussis*-titers in the nasal lavage fluid from both IN-aP and IN-caP vaccinated groups, although only IN-aP resulted in a significant increase compared to baseline levels (Fig. 4b). The presence of *B. pertussis* binding IgA in the lungs and nasal cavity suggests that IN DTaP is capable of priming a mucosal immune response in the upper and lower murine respiratory systems.

### Intranasal immunization decreased pulmonary pro-inflammatory environment

**Fig. 5:**
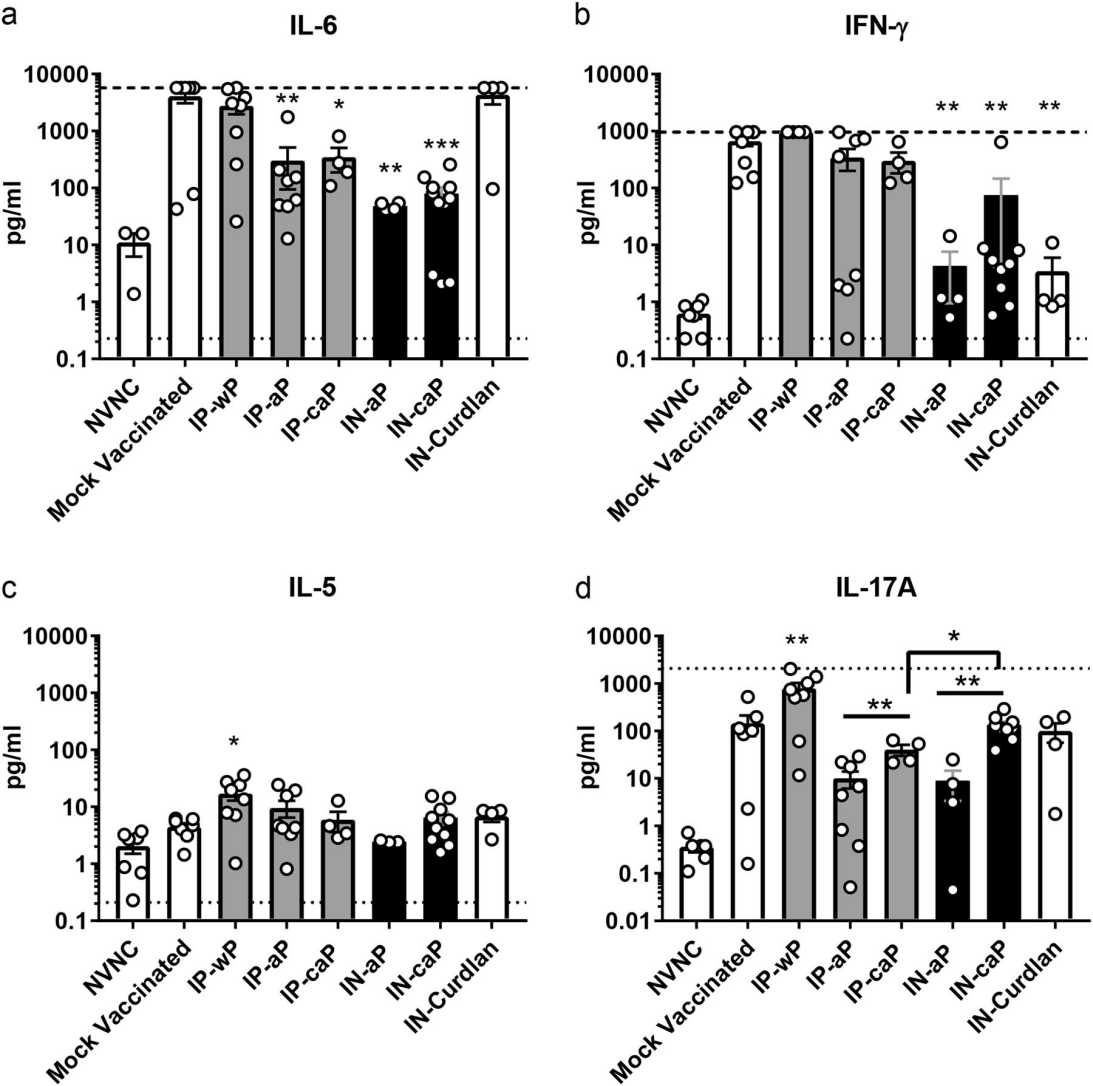
Intranasal immunization decreased pulmonary pro-inflammatory cytokines during challenge. Analysis of cytokines from supernatant of lung homogenate at day 3 pc. Cytokines **a** IL-6, **b** IFN-γ, **c** IL-5, **d** IL-17A were quantified by electrochemiluminescence assay. Results shown as mean ± SEM (*n* = 4–8), *P *<* 0.05, **P *<* 0.01, *** *P* *<* 0.001, ****P *<* 0.0001. *P* values were determined by one-way ANOVA with Dunnett’s post hoc test compared to mock vaccinated mice. Bars connecting groups indicate values determined by two-tailed un-paired *t*-test. Upper and lower limits of detection shown as dash or dotted lines, respectively if data points reached these limits

It has been well established that neutralization of PT and inhibition of bacterial adhesins associated with DTaP protection leads to a markedly reduced pro-inflammatory environment at the site of infection when compared to a natural infection.^17^ Conversely, challenge in whole-cell protected animals resulted in a severe pro-inflammatory response, similar to the natural infection of *B. pertussis*.^19^ We hypothesized that the immunostimulatory properties of curdlan would induce a more pro-inflammatory response following challenge with *B. pertussis* compared to IP-aP. Cytokine concentrations were determined from supernatant of lung homogenate at day 3 pc. The pro-inflammatory interleukin-6 (IL-6) has been shown to be increased following *B. pertussis* infection of a naive mouse, while DTaP induced protection drastically decreases IL-6 production.^42,44^ Our results demonstrate a significant reduction of IL-6 in the lungs of either IN-aP or IN-caP immunized mice when compared to mock vaccinated or IN-curdlan control mice, and are comparable to levels observed in IP immunized groups (Fig. 5a). We measured a similar reduction in the Th1 associated cytokine IFN-γ in the IN administered groups, IN-aP and IN-caP; although, these levels were higher in IN-caP immunized mice (Fig. 5b). We only observed a significant increase in the Th2 associated interleukin-5 in IN-wP immunized mice compared to mock vaccinated mice (Fig. 5c)

IN administration of curdlan, and moreover, vaccine administration through the IN route regardless of adjuvant has been shown to induce an increased IL-17 response.^30,32^ Thus, we hypothesized that we would observe increased levels of IL-17 in mice immunized with IN-caP compared to IN-aP or IP-aP. We quantified IL-17A in the lung supernatant and observed significant increases of IL-17A with the addition of curdlan in IP-caP and IN-caP immunized mice compared to IP-aP and IN-aP groups, 4-fold and 14.9-fold respectively (Fig. 5d). Furthermore, we observed that IN administration induced a significant increase in IL-17A compared to IP immunization. This IL-17A response was lower than the robust IL-17A induced by IP-wP (Fig. 5d).

**Fig. 6:**
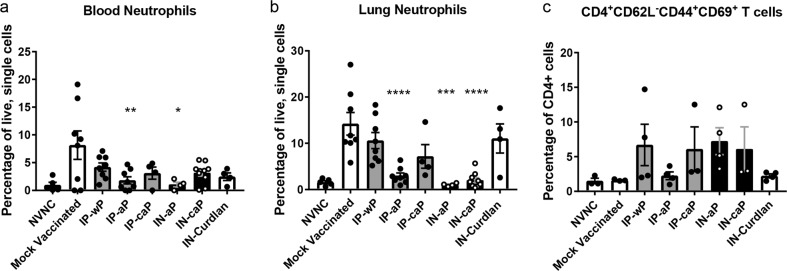
Intranasal immunization reduced neutrophil accumulation in the lung and circulating neutrophils, but did not generate lung T_RM_ population after *B. pertussis* challenge. **a** The percentage of live, CD11b^+^Gr-1^hi^ neutrophils from a single cell suspension of the peripheral blood. **b** The percentages CD11b^+^Gr-1^hi^ neutrophils in single cell lung homogenates. **c** The percentage of CD4+ T cells that are CD62L^-^CD44^+^CD69^+^ isolated from the lung at day 3 pc. Results shown as means ± SEM, *P < 0.05 **P < 0.01, ***P < 0.001, ****P < 0.0001 (*n* = 4–8). *P* values were determined by one-way ANOVA with Dunnett’s post hoc test compared to mock vaccinated mice

Natural infection with *B. pertussis* is known to cause severe leukocytosis in the murine model,^45^ which can be measured by elevated neutrophils in the peripheral blood. Following *B. pertussis* challenge, we observed that all vaccinated groups ameliorated symptoms of leukocytosis by day 3 pc; however, only the administration of DTaP either by IP or IN administration significantly reduced CD11b^+^Gr-1^hi^ neutrophils in the peripheral blood (Fig. 6a). In the lungs, neutrophils were decreased in IN-aP, IN-caP, and IP-aP immunized groups compared to mock vaccinated mice.

Due to the expansion of T_RM_ in the lungs observed by others following administration of parenteral and intranasal administered pertussis vaccines, we aimed to determine if IN-aP or IN-caP could induce the expansion of this population. CD4 T cells were isolated from the lung at day 3 pc, and were identified as T_RM_ cells based on expression of surface markers: CD4 + CD62L-CD44 + CD69+. We did not observe a statistical difference of this population following challenge with either IP or IN administered vaccines; although, we did observe a slight increase in IP-wP, IP-caP, IN-aP, and IN-caP compared to mock vaccinated mice (Fig. 6c). Taken together these data suggest that immunization with DTaP through the IN route reduces the pro-inflammatory environment of the murine lung during *B. pertussis* challenge in a manner similar to IP-aP-mediated protection.

### Intranasal immunization reduced respiratory *B. pertussis* bacterial burden

**Fig. 7:**
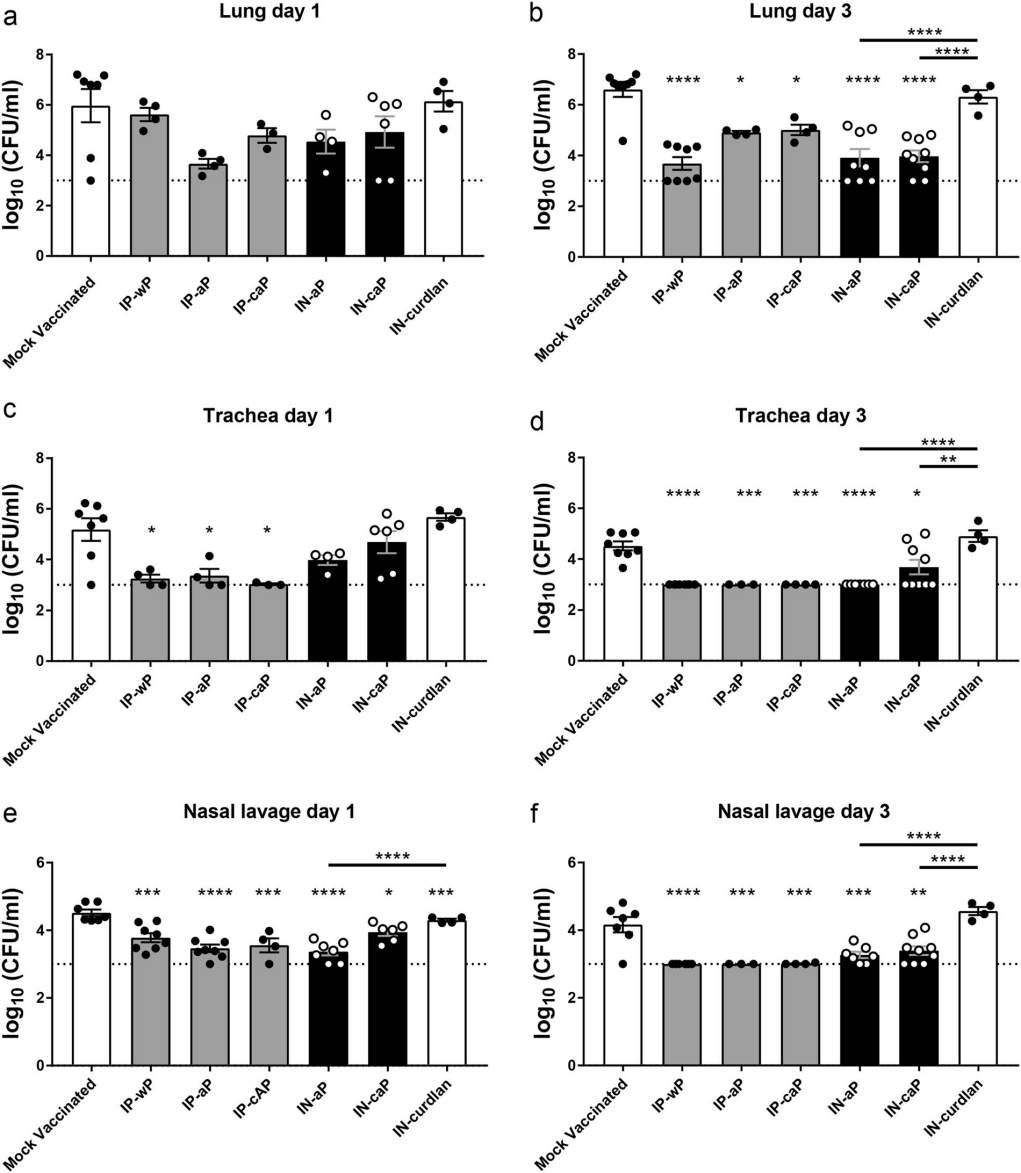
Intranasal immunization reduced respiratory *B. pertussis* bacterial burden. Analysis of bacterial burden was determined at days 1 and 3 pc. Bacteria were quantified by counting of serially diluted CFUs following immunization and challenge. CFU counts were determined from lung homogenate (**a**, **b**), trachea homogenate (**c**, **d**), and nasal lavage fluid (**e**, **f**). Results are mean ± SEM (*n* = 4–8, with four averaged technical replicates) from two independent experiments. *P *<* 0.05, **P *<* 0.01, ***P *<* 0.001, ****P *<* 0.0001. *P* values were determined by one-way ANOVA with Tukey’s post hoc test compared to mock vaccinated mice, or between connected columns. The dashed line represents the lower limits of detection due to plating

Lastly, we examined the clearance of *B. pertussis* from the respiratory tract following IN immunization. At days 1 and 3 pc, viable bacterial burden was quantified by counting of CFU in the lung, trachea, and nasal lavage fluid. We observed a significant reduction in viable bacteria recovered from the lung in all immunized groups by day 3 pc; however, these changes were not observed at day 1 pc (Fig. 7a, b). IN-aP and IN-caP immunized mice bacterial burdens were reduced by 99.4% and 99.7%, respectively, compared to mock vaccinated mice. This reduction in viable bacterial burden was similar to that of mice immunized by IP-aP, an immunization that is known to be effective (Fig. 7b). This reduction in bacterial burden was not observed following immunization with the negative control (IN-curdlan), suggesting an antigen-specific response. Similar trends were observed in the trachea homogenate (Fig. 7c, d), and nasal lavage fluid (Fig. 7e, f), as all immunized groups regardless of IP or IN delivery were significantly reduced compared to mock vaccinated mice. In summary, we observed similar clearance of *B. pertussis* from the respiratory tract of mice immunized IN compared to mice immunized with vaccines known to be protection delivered by the IP route.

## Discussion

The natural immune response to *B. pertussis* is initiated by the colonization of the respiratory tract, which leads to a cascade of inflammatory cytokines such as IL-6, IFN-γ, TNF-α, and IL-1β leading to increased inflammatory infiltrate consisting of: dendritic cells, macrophages, neutrophils, and lymphocytes.^42,46^ Numerous studies suggest the infection is cleared through opsonization of bacteria by the humoral immune response, combined with a Th1/Th17 cell-mediated response.^15,19,47,48^ Additionally, factors associated with a local immune responses have been observed in convalescent mice following challenge with *B. pertussis*, such as antigen-specific IgA titers and T_RM_ cells, which were shown to decrease bacterial burden when adoptively transferred to naive mice.^21^

The induction of a protective immune response also has been observed through mucosal immunizations against *B. pertussis*. Oral vaccination with heat-killed *B. pertussis* in newborn infants resulted in the production of *B. pertussis* specific serum and mucosal antibodies; although, this protection was not long lived.^49^ In the mouse model, a live-attenuated IN pertussis vaccine, BPZE1, has shown protection up to one year after immunization,^50^ and is in clinical trials.^51^ Furthermore, BPZE1 vaccine produced antigen-specific IgA antibodies in the baboon model, indicative of elicitation of mucosal immune response.^52^ The mucosal immune response was further explored in a murine model where BPZE1 induced a protective secretory IgA immune response, and the generation of long lasting IL-17 secreting T_RM_ cells in the respiratory system.^24^ Similarly, this population of antigen-specific memory cells was found to be expanded by an acellular pertussis vaccine (LP-GMP) consisting of a TLR2 agonist and intracellular receptor STING agonist, which correlated with the IL-17 secreting T_RM_ population.^23^ Promisingly, generation of the IL-17 secreting T_RM_ cell population was observed following whole-cell immunization through IP administration; however, Tdap administration through the same route did not generate this population.^20^

In our study, we demonstrate that by altering the administration route of DTaP immunization from IP to IN delivery, the vaccine still induced a protective immune response. Using fluorescent labeled DTaP particles we visualized that IN-aP was readily detectable (Fig. 1). In vivo imaging analysis demonstrated significantly more labeled IN-caP present in the nasal cavity of mice. Further work examining the lymph nodes responsible for antigen priming would be beneficial to determine the role of nasal associated lymphoid tissue compared to vaccination of the lung, and trafficking to mediastinal lymph nodes. The location of antigen priming following IN immunization may play a critical role in the optimization of future human IN vaccines. Determining if antigen uptake in the nasal cavity is essential to induce a protective immune response would play a key role in the development of a human IN pertussis vaccines.

We determined that while IN-caP vaccinated mice had significantly lower detection of DTaP in the lungs compared to IN-aP immunized mice that the bacterial burden was similar between the two groups (Fig. 7), suggesting that antigen priming still occurred. This was further demonstrated through generation of robust antigen-specific serum IgG antibody titers from the two IN immunized groups (Fig. 3). The IgG subclass data (Fig. 3) suggest that immunization with IN-aP most likely induces a Th2 response. Other studies with BPZE1 or LP-GMP adjuvants have observed shifts to Th1 response using IN route.^20,24^ Additionally, in these studies the authors did not observe a significant reduction in bacteria burden of the nasal cavity of mice parenterally immunized with acellular pertussis. However, in the current study we observed contradictory results suggesting significant reductions in nasal bacterial burden after IP-aP and IP-caP immunizations. The current study was performed using *B. pertussis* strain UT25 with the challenge dose of 2 × 10^7^ CFUs administered by intranasal inoculation in 20 µl. The inoculum dose is higher than previous mentioned studies, and those studies were also performed using different *B. pertussis* strains and mouse strains. Another difference that we believe is most likely accounting for the discrepancies, could be the different methods of culturing viable bacteria from the nasal cavity resulting in lower limits of detection. The aforementioned studies and our study support the idea that IN immunization can be protective, and further development should be pursued.

A key finding of our current study was the production of antigen-specific IgA in the respiratory system only in mice that were administered DTaP through IN administration: IN-aP and IN-caP (Fig. 4). Another difference between the IN-aP and IN-caP groups was the level of IL-17A present in the lung homogenate (Fig. 5). We found that inclusion of curdlan in DTaP resulted in higher levels of IL-17A whether administered through IN or IP route although IL-17A was highest in IN-caP. These findings correlate with results from others suggesting a role of curdlan and IN administration in IL-17 production.^30,32^ It is clear that other formulations of intranasal pertussis immunizations are capable of inducing a robust IL-17 response, specifically in the respiratory tract, although in our current study the level of IL-17A production in lungs was lower than other vaccine formulations.^23,24^ Likewise, we did not observe an increase of T_RM_ cells present in the lung that has been demonstrated by the same groups.^22–24^ There is a clear role of IL-17 secreting T_RM_ cells in the immune response against pertussis.^21–23,25,53^ It may be possible that the increases of IL-17A observed in the lungs of IP-caP, IN-aP, IN-caP immunized groups are related to the slight expansion of lung T_RM_ cells; however, further studies are needed to show that correlation. It is known that live-attenuated vaccines are more potent inducers of T_RM_ populations.^54^ Taking into account the pro-inflammatory response to challenge following administration of wP, BPZE1 and LP-GMP^22–25^ and the lack of inflammation observed following challenge of mice immunized with IN-aP and IN-caP, it is conceivable that IN-aP and IN-caP are not immunomodulatory enough to induce a strong T_RM_ response. This work demonstrates induction of mucosal immune response specific to *B. pertussis* in the respiratory system but, future work is needed to identify the duration of protection.

In our model, addition of curdlan to DTaP increased IL-17 responses but did not improve clearance (Fig. 7). A caveat of the current work is the role of aluminum hydroxide was not addressed. All IN immunizations consisted of DTaP, which contains aluminum hydroxide, therefore, observations from IN-caP may be affected by the presence of two adjuvants. We hypothesized that the addition of curdlan adjuvant into the DTaP formulation would skew the immune response to a Th1/Th17 cell-mediated response. The combination of multiple adjuvants paired to aluminum has been explored previously, leading to the retention of antigens at the injection site with the new benefit of recruiting APCs to the site.^55^ The combination of MPLA with aluminum hydroxide has been shown to increase antibody titers and shift the immune response from Th2 to Th1 associated cytokine production from spleen cells in vaccines for hepatitis B and human papilloma virus.^56^ Likewise, the addition of CpG oligonucleotides with aluminum hydroxide into a hepatitis B vaccine enhanced the antibody response by shifting from IgG1 to IgG2a antibodies in mice.^57^ In the case of these two vaccines aluminum hydroxide and the adjuvants, MPLA and CpG induce protection through mechanisms not shared by aluminum. As mentioned, curdlan as a vaccine adjuvant is known to promote a Th1/Th17 skewed responses.^30,58^ It has been demonstrated that curdlan binds to dectin-1 inducing not only the canonical activation of NF-κB subunits p65 and c-Rel, but also the non-canonical subunit RelB through the spleen tyrosine kinase in human DC cells.^59^ Gringhuis et al. measured NF-κB activation through Raf-1 kinase independent of Syk following exposure to curdlan.^31^ Furthermore, the authors demonstrate Raf-1 activation suppressed Syk-induced RelB promoting the Th1/Th17 polarizing cytokines. Through this mechanism, Raf-1 activation induced by curdlan modulated TLR-2 and TLR-4 induced cytokine expression.^31^ It may be possible that dectin-1 cross-talk with other PRRs is interfering with aluminum hydroxide-induced response. For instance, damage to phagolysosomes following the phagocytosis of aluminum has been shown to activate the Syk signaling pathway, which is shared by dectin-1. Additionally, both curdlan and aluminum hydroxide are known to activate the NLRP3 inflammasome.^60,61^ Future studies will be required to elucidate potential interference between adjuvants, as these interactions may be limiting the immunomodulatory actions necessary to induce the expansion of T_RM_ and IL-17 response in the respiratory tract. Future experimental vaccines with aluminum hydroxide or curdlan will be compared with only one adjuvant in each formulation. Alum is not used in any currently licensed nasal vaccines and curdlan is not an approved adjuvant for use in humans. Mucosal pertussis vaccines would require appropriate safety evaluation to move forward for clinical development.

In conclusion, we observed that by altering the route of administration from IP to IN a protective response was reproduced, resulting in clearance of the pathogen to limits of detection. Furthermore, we determined that IN delivery of DTaP was sufficient to prime a local mucosal immune response at site of infection. The inclusion of the adjuvant curdlan in DTaP immunization did increase IL-17 production in the lung; however, we did not observe a shift in the type of immune response compared to DTaP alone. In humans, DTaP and Tdap do not provide perfect protection against pertussis; however, there is no clear path forward to the next generation vaccine. It is clear that developing a new vaccine will take considerable efforts. Here in our study we observed a surprising result, that IN DTaP vaccine could protect mice against *B. pertussis*. These data cause us to wonder if a change in the route of DTaP immunization would be a promising booster vaccine strategy.

## Methods

### Composition and administration of vaccines used in study

The vaccines administered in the study were prepared no longer than 1 h before administration. INFANRIX (GSK) human vaccine (DTaP), and the National Institute for Biological Standards and Control WHO whole-cell pertussis vaccine (NIBSC code 94/532) were used in the study. Vaccines were diluted with PBS to 1/12th of the human dose (based on total antigen content). Curdlan adjuvant was administered at 200 µg per mouse. A vaccine dose of 1/12th the human dose was the highest concentration of vaccine that was possible to use due to volume of vaccine required for the solubility of curdlan. Curdlan (Invivogen, tlrl-curd) was prepared by dissolving 50 mg in 2.5 ml sterile purified water. Curdlan preparation was brought into solution by adding 100 µl 1 N NaOH and vortexing. The curdlan suspension (20 mg/ml) was then sonicated for 10 min and placed in 37 °C water bath until administration. At the time of immunization, the vaccines containing curdlan were administered in a liquid form to reduce risk of choking due to excessive gel formation in the airway. IN administered mice recovered at the same rate of IP immunized mice. These experiments were conducted in accordance with the National Institutes of Health Guide for the care and use of laboratory animals. The protocols used were approved by West Virginia University Institutional Animal Care and Use Committees (WVU-ACUC protocol 1602000797).

### Vaccination of mice for vaccine particle tracking

CD-1 (outbred; strain code 022) mice aged four weeks were obtained from Charles River Laboratories. At five weeks mice were anesthetized with 77 mg/kg ketamine and 7.7 mg/kg xylazine. Mice were administered 50 µl of vaccine or control, with 25 µl into each nostril (IN).

### Tracking of DTaP in respiratory system

DTaP vaccine particles were labeled using the Alexa Fluor 660 Protein Labeling Kit (Molecular Probes). Briefly, 0.5 ml of DTaP vaccine was added to 50 µl of 1 M sodium bicarbonate, then added to Alexa Fluor 660 dye stock. The mixture was incubated for 1 h at room temperature with agitation. The solution was concentrated by dialysis in phosphate-buffered saline overnight to remove unlabeled dye. Vaccine particles were examined using the Cy5 channel on an EVOS FL microscope. Particles were mounted on a slide and visualized using a 100X objective. Particle diameter was measured using ImageJ (version 1.52a) with the line segment tool in proportion to the scale bar. Four fields of view were measured to determine particle size and standard deviation. Labeled vaccine was used to immunize mice. At 0, 6, 12, and 24 h post vaccination fluorescent signal was measured using an IVIS Spectrum (Xenogen). Mice were anesthetized using 3% isoflurane, mixed with oxygen prior to and throughout imaging. The following parameters were used: (1) A binning setting of 4 was kept constant for all images, (2) each image was quantified using the automatic image setting, (3) fluorescence photons were measured using total radiant efficiency of a common region of interest placed on the nasal cavity, and (4) measurements were normalized using Living Image (Xenogen ver. 2.5). Use of this quantification method accounted for variations in exposure between images.

At 6, 12, 24, and 48 h post vaccination, animals were euthanized to quantify DTaP in lungs by flow cytometry analysis. Lungs were removed, and homogenized using gentleMACS C tubes (Miltenyi Biotec) with enzymatic lung dissociation kit (Miltenyi Biotec, 130-095-927). All samples were blocked using Fc Block (BD), then labeled with Alexa Fluor 700-conjugated CD11b (Biolegend, 101222), DTaP particles were detected with Cy5 channel. Following a 1 h dark incubation labeled samples were washed, then fixed using 0.4% w/v paraformaldehyde overnight. Samples were resuspended in PBS and analyzed on a LSR Fortessa flow cytometer (BD). DTaP containing myeloid cells were classified as CD11b^+^DTaP^+^ single, live cells.

### Detection of DTaP particles in lung and nasal cavity

Detection of DTaP particles in the lung and nasal cavity were confirmed using confocal imaging. Mock vaccinated and challenged mice were euthanized at 6 h post vaccination. Prior to homogenization, the post-caval lobe of the mouse lung was removed. The post-caval lobe was flash frozen in OCT medium (Tissue Plus, Fisher Healthcare), using liquid nitrogen. The samples were stored at −80 °C until sectioning. Sectioned samples (6 µm) were fixed in acetone, then stained with ActinGreen Ready Probes (Invitrogen) and NucBlue Ready Probes (Invitrogen), using manufacturer protocols.

Skulls were removed from mouse, and the lower jawbone discarded. The skulls were fixed in formalin for 12 h at 4 °C, then de-calcified at room temperature for 24 h, before samples were embedded in paraffin. Sectioned samples were de-paraffinized and rehydrated using xylene, and washes with decreasing ethanol concentrations (100–70%). An antigen retrieval step was performed using citrate buffer, where samples were heated to 98 °C for 20 min. Samples were then stained as mentioned above. Samples were analyzed for DTaP particles in tissue and airway mucus using a Nikon A1R confocal microscope. Images were acquired using DAPI, FITC, and Cy5 channels using a 100× oil immersion lens (100×/1.40 Nikon Plan Apo). DTaP particles were quantified using ImageJ. Briefly, the threshold tool was used to select only the fluorescent particles above background levels. Then, the threshold adjusted area was quantified using the analyze particles tool. Thus, the data is represented as the percentage of fluorescent particles per area of the total image field. Three image fields per sample were quantified and averaged per mouse.

### *B. pertussis* strains and growth conditions

*B. pertussis* strain UT25Sm1^62^ was used for murine challenge in all experiments. UT25Sm1 was cultured^42^ on Bordet Gengou agar plus 15% defibrinated sheep’s blood (Remel) with streptomycin 100 mg/ml. *B. pertussis* was incubated at 36 °C for 48 h, then transferred to modified Stainer-Scholte liquid medium,^63^ without the cyclodextrin, heptakis. Liquid cultures were incubated for 24 h at 36 °C, with shaking until reaching an OD_600_ of ~0.6, at which time cultures were diluted for challenge dose.

### Vaccination and *B. pertussis* challenge

IN immunized mice received 50 µl of vaccine as described above. IP immunized mice received 200 µl of vaccine injected into the peritoneal cavity. IN and IP immunized mice received the same antigen dose of 1/12th. Mice received a boost of the vaccines with the same concentrations twenty-one days after initial immunization. At thirty-five days post initial vaccination, mice were challenged with 2 × 10^7^ CFU *B. pertussis* administered in 20 µl through nostrils. At days 1 and 3 pc mice were euthanized, blood and respiratory tissue were isolated as previously described.^42^ Peripheral blood was collected by cardiac puncture following euthanasia. Trachea and lungs were removed and separately homogenized using Dounce homogenizers to determine viable bacteria burden in respiratory tissue. A nasal cavity lavage was performed using 1 mL of PBS flushed through the nares to determine bacterial burden of nasal cavity. Serial dilutions in PBS were plated on BG containing streptomycin (100 µg/ml) to ensure that only UT25Sm1 *B. pertussis* was cultured

### Serological analysis of *B. pertussis* specific antibodies

Serological responses specific to *B. pertussis* antigens were quantified by ELISA. High-binding microtiter plates were coated with PT (50 ng/well)(LIST Biologicals) and FHA (50 ng/well)(Enzo Life Sciences). Serological responses against UT25Sm1 were cultured to an OD_600_ of 0.24 and microtiter plates coated with 50 µl of bacteria per well. Bound antibodies were detected using goat anti-mouse IgG (1030-04), IgA (1040-04), IgG2a (1081-04), and IgG1 (1071-04) antibody conjugated to alkaline phosphatase (1:2000) (Southern Biotech). Positive antibody titers were determined using a baseline set at two times the average of blanks.

### Quantification of pulmonary and blood APCs

To determine cell types infiltrating the lung and leukocytes present in peripheral blood, single cell suspensions from the tissues mentioned above were prepared, as described previously.^42^ Briefly, lung tissue was homogenized by Dounce homogenizers, filtered with a 100 µm filter, and red blood cells were lysed for 2 min at 37 °C (Pharmlyse). Single cell populations were blocked by initial incubation with Fc Block (BD, 553141) for 15 min at 4 °C. Cell populations were incubated in the dark with antibodies to cell surface markers for 1 h at 4 °C. Neutrophil populations were identified using: PE-conjugated GR-1 (BD, 553128,1:500), Alexa Fluor 700-conjugated CD11b (Biolegend, 101222, 1:500). Neutrophils were classified as CD11b^+^Gr-1^hi^ single, live cells. TRM populations were determined using: APC-Cy7-conjugated CD4 (Biolegend, 100526, 1:500), BB515-conjugated CD44 (BD, 564587, 1:500), APC-conjugated CD62L (BD, 553152, 1:500), and BV421-conjugated CD69 (BD, 562920, 1:500). The TRM gating strategy was adapted from Wilk et al., shown in Supplementary Fig. 1.^21^

### Lung homogenate cytokine analysis

To quantify inflammatory cytokines at the site of infection, lung homogenate samples were centrifuged at 19 × *g* for 4 min supernatant was removed and stored at −80 °C. Quantitative analysis of cytokines was performed using Meso Scale Discovery cytokine kits: V-PLEX pro-inflammatory panel (K15048D) and IL-17A V-PLEX (K152RFD), per the manufacturer’s instructions.

### Statistical analysis

Experiments in the study were performed with 3 to 8 biological replicates. Data were analyzed using GraphPad Prism 7. ROUT method was used to removed outliers.^64^ Comparisons between groups were performed using one-way analysis of variance (ANOVA) with Dunnett’s and Tukey’s post hoc tests. Comparisons between groups with or without curdlan were analyzed by two-tailed unpaired *t*-test, when applicable multiple *T*-tests with Holm-Sidak post hoc test were applied to curdlan inclusion groups.

### Reporting summary

Further information on research design is available in the Nature Research Reporting Summary linked to this article.

## Supplementary information

Supplemental Figure 1

Reporting Summary

## Data Availability

Data for all figures are available upon reasonable request to the corresponding author.
