# Supplementary material for: Intranasal acellular pertussis vaccine provides mucosal immunity and protects mice from Bordetella pertussis
Source: NPJ Vaccines. 2019 Oct 3;4:40. doi: 10.1038/s41541-019-0136-2 (PMC6776550; doi:10.1038/s41541-019-0136-2)
Supplement: Supplementary file 1 — Supplemental Figure 1 [file 41541_2019_136_MOESM1_ESM.pdf]

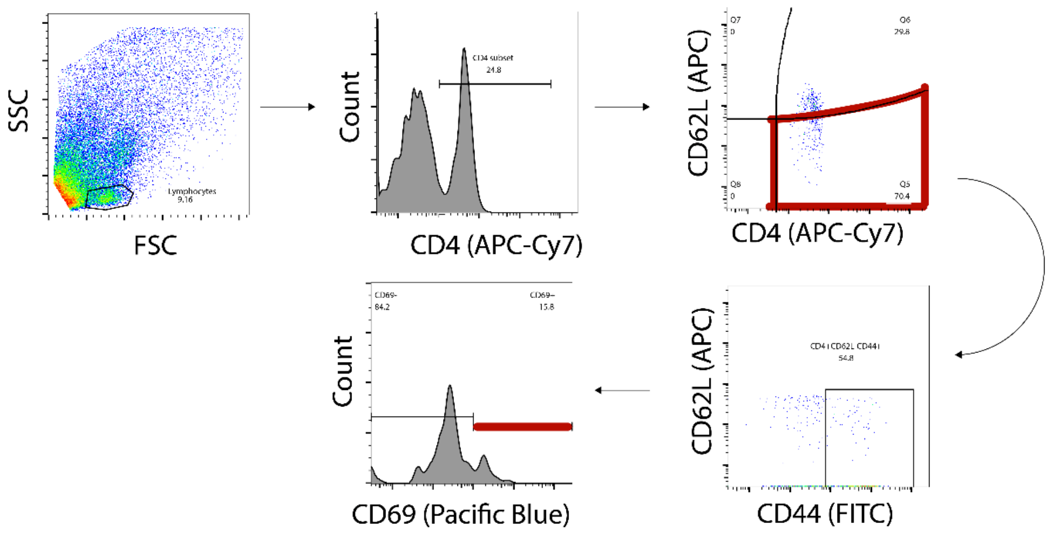

Supplementary Figure 1. Gating strategy used to isolate $CD4^+CD62L^-CD44^+CD69^+$ T cells. Live, single lymphocytes were gated based on size and granularity using FSC and SSC. T cells were determined by CD4 expression. $CD62L^-CD4^+$ cells, were gated on the T cell activation marker CD44. Finally, $CD62L^-CD4^+$ $CD44^+$ cells were gated on expression of the tissue homing marker CD69. These data are represented in Fig. 4c.
